# Circulating VEGF and inflammatory bowel disease: a bidirectional mendelian randomization

**DOI:** 10.3389/fgene.2024.1282471

**Published:** 2024-04-18

**Authors:** Haishan Lin, Bangwei Cao

**Affiliations:** Cancer Centre, Beijing Friendship Hospital, Capital Medical University, Beijing, China

**Keywords:** VEGF, IBD, GWAS, SNP, mendelian randomization

## Abstract

**Introduction:** Prior observational studies have suggested an association between circulating vascular endothelial growth factor (VEGF) levels and inflammatory bowel disease (IBD). This study sought to demonstrate the directionality of the association between circulating VEGF and particular forms of IBD as well as if there is a causal relationship between them.

**Methods:** We collected summary data from relevant genome-wide association studies (GWASs) to assess the validity of causality, and a two-sample bidirectional Mendelian randomization (MR) study and sensitivity testing were performed to assess the causal relationship between circulating VEGF and IBD risk, including Crohn’s disease and ulcerative colitis.

**Results:** Our findings revealed a direct causal link between circulating VEGF and Crohn’s disease (b 0.195, se 0.078, *p* < 0.013). However, neither circulating VEGF nor ulcerative colitis were shown to be causally linked (*p* > 0.025), nor was there proof of a reverse causal relationship from IBD to VEGF.

**Discussion:** In conclusion, circulating VEGF shows a cause-and-effect relationship with Crohn’s disease.

## Introduction

The process of creating new blood vessels, known as angiogenesis, has been identified as a key component in the pathogenesis of chronic inflammatory disorders, with its involvement exacerbating disease progression (Chidlow et al., 2007). Angiogenesis, the formation of new blood vessels from existing ones, is a crucial process in various physiological and pathological conditions. The original text highlights the importance of several well-known angiogenic factors such as vascular endothelial growth factor (VEGF), angiopoietins, platelet-derived growth factor, transforming growth factor-β, and basic fibroblast growth factor. Recent investigations have shed light on the significant roles of Yes-associated protein 1 (YAP) and transcriptional co-activator with PDZ-binding motif (TAZ) in promoting angiogenesis (Lee et al., 2021). Vascular Endothelial Growth Factor (VEGF) indeed takes the spotlight as a major contributor to angiogenesis when compared to other proangiogenic factors. Its pivotal role in promoting the growth of new blood vessels makes it a key target for medical research and therapeutic development. Understanding VEGF’s prominence in angiogenesis provides critical insights for devising treatments, especially in conditions where angiogenesis is a central factor, such as cancer and tissue regeneration (Eichholz et al., 2010). Five glycoproteins, including VEGF-A, VEGF-B, VEGF-C, VEGF-D, and VEGF-E, as well as two placental growth factors, PLGF-1 and PLGF-2, are members of the VEGF-associated gene family (Ferrara et al., 2003). VEGF-A, sometimes referred to as VEGF, is essential for angiogenesis, playing a critical role in both pathological and physiological contexts (Grothey and Galanis, 2009). In the context of inflammatory bowel disease (IBD), VEGF has garnered attention due to its observed overexpression (Kanazawa et al., 2001; Di Sabatino et al., 2004). This has led to speculations that IBD may have a unique mediator in the form of VEGF-A, as this molecule appears to stimulate angiogenesis and inflammation in the intestinal tract (Franco et al., 2009). Notably, besides neoangiogenesis and vascular injury, IBD is also associated with increased lymphangiogenesis (Simon and Detmar, 2019). A study has highlighted the involvement of lymphatics in the development of intestinal inflammation, suggesting that VEGF-C might hold potential in restoring impaired lymphatic function and therefore presenting a potential therapeutic avenue for IBD management (D'Alessio et al., 2014). Nevertheless, the precise role of soluble VEGF (sVEGF) elevation, whether it acts as a mere side effect or has a direct contribution to IBD pathophysiology, remains uncertain. To completely understand the complex processes behind the role of VEGF and its related variables in the onset and progression of IBD, more research is necessary. In the context of IBD, the overexpression of VEGF and the involvement of lymphatics further highlight its importance. However, additional studies are needed to completely unravel the complex interplay between VEGF and IBD, particularly regarding the impact of sVEGF elevation.

In this investigation, we used publically available genome-wide association study (GWAS) summary statistics to carry out a two-sample bidirectional Mendelian randomization (MR) analysis. MR is a potent technique that examines causal links between exposures and outcomes by using genetic variations as instrumental variables (IVs) (Smith and Ebrahim, 2003). We may deduce the causal relationship between Vascular Endothelial Growth Factor (VEGF) and the risk of inflammatory bowel disease (IBD) by utilizing the Mendelian inheritance principle, which guarantees that genetic variations are randomly distributed at conception. The use of IVs helps overcome confounding biases that are commonly encountered in traditional epidemiological studies. Our study design accounts for potential reverse causation, where the timing of exposure and outcome is reversed. This is crucial in establishing a robust causal relationship. By using IVs, we can minimize the impact of reverse causation and obtain more reliable results. The findings from our two-sample bidirectional MR analysis will provide valuable insights into the connection between VEGF and the risk of IBD. This may help with a better comprehension of the underlying mechanisms and potentially guide future interventions and treatments for IBD.

## Materials and methods

### GWAS statistics of vascular endothelial growth factor

A thorough GWAS meta-analysis of circulating cytokines and growth factors was used to obtain summary data for circulating VEGF (Folkersen et al., 2020). These figures were gathered from GWAS of the Olink CVD-I proteins from 13 cohorts of people with European ancestry, totaling 21,758 people. The information on all research cohorts is included in Supplementary Table S1. Age, sex, body mass index, and the top 10 genetic main components were all taken into account when adjusting the GWAS statistics. Single nucleotide polymorphisms (SNPs) related to circulating VEGF were included as the exposure and VEGF was also considered as an outcome in our bidirectional MR investigation. As soon as it was determined that VEGF may have a plausible causal influence on particular cancer types, we revalidated the results using VEGF as exposure from a separate GWAS (Folkersen et al., 2017).

### GWAS statistics of different types of IBD

The IEU OpenGWAS (MR Base) public database (https://gwas.mrcieu.ac.uk/) provided summary statistics for a number of malignancies. Taking into account the publicly accessible summary information, genetic variations linked to Crohn’s disease (ieu-a-12) and ulcerative colitis (ieu-a-970) were taken out (Liu et al., 2015). People of European heritage were included in this study since two independent samples from the same group were needed for two-sample MR. We studied the reverse causal inference using the same MR techniques when a probable causal impact of VEGF on IBD was identified, with IBD serving as the exposure and VEGF serving as the result. The summary statistic on Crohn’s disease was obtained from a large GWAS, which encompassed whole-genome sequencing data for 17,897 Crohn’s disease cases and 33,977 controls. Similar to this, a significant GWAS that included whole-genome sequencing data for 13,768 ulcerative colitis patients and 33,977 controls was used to obtain the summary statistic for ulcerative colitis. The majority of the population under study was European.

### Mendelian randomization statistical analysis

To find out if certain IBDs are at increased risk due to circulating VEGF, conducted a forward MR analysis. We selected SNPs closely correlated with VEGF from GWAS results. In this analysis, VEGF served as the exposure, while Crohn’s disease and ulcerative colitis functioned as the outcomes. Conversely, to examine if IBDs led to an increase in circulating VEGF, we conducted a reverse MR analysis, selecting SNPs related to Crohn’s disease or ulcerative colitis as IVs. In this case, IBDs were the exposures and VEGF was the outcome.

To qualify as IVs, SNPs associated with the exposure had to fulfill three criteria: genome-wide significance (*p*-value <5 × 10⁻⁶ for forward analysis; *p*-value <5 × 10⁻⁸ for reverse analysis), minor allele frequency >0.01, and no linkage disequilibrium (r^2^ = 0.01 and KB = 10,000). To calculate the impact of specific SNPs connected to the exposure on the result, we used the Wald ratio approach. Subsequently, we pooled the impact magnitude of each SNP using the inverse variance weighting (IVW) technique. For the MR statistical analysis, the weighted median and MR-Egger procedures were complimentary approaches (Burgess et al., 2016; Burgess and Thompson, 2017a). Heterogeneity and horizontal pleiotropy were evaluated using Cochrane’s Q value and MR-Egger intercept (Burgess and Thompson, 2017b), respectively. In case of evidence for horizontal pleiotropy, we applied the MR-PRESSO outlier test (Verbanck et al., 2018), which uses the IVW approach as its foundation to filter out anomalies and calculate causal effects. The IVW approach was regarded as the major assessment method in the absence of heterogeneity and horizontal pleiotropy. The use of the multiplicative random effects IVW method in genetic studies is essential for drawing accurate causal inferences. When both IVW and weighted median analyses point in the same direction, it reinforces the credibility of the causal influence in the presence of genetic heterogeneity. The weighted median estimate is particularly valuable because it maintains reliability even in situations where a significant proportion, up to 50%, of the genetic instrumental variables (IVs) may be invalid (Bowden et al., 2016). This stands in contrast to the IVW approach, which necessitates that all single nucleotide polymorphisms (SNPs) employed as IVs are valid. This robustness of the weighted median method is especially advantageous when working with large datasets, where some genetic instruments may not be perfectly reliable. It allows researchers to draw meaningful conclusions even in the face of potential instrument weaknesses, enhancing the overall robustness of causal inference in genetic research.

In order to do a sensitivity analysis, we built funnel plots and did a leave-one-out analysis. We tested whether estimates were entirely influenced by a particular SNP using a leave-one-out approach. Conducting a sensitivity analysis is a crucial step in ensuring the robustness of research findings. In this process, funnel plots and leave-one-out analyses serve as valuable tools. Funnel plots provide a visual representation of the distribution of SNP representation points and can reveal potential biases or outliers in the data. Ideally, in the absence of heterogeneity, these points should exhibit a symmetrical distribution, indicating a balanced and unbiased dataset. The leave-one-out approach, on the other hand, helps assess the influence of individual SNPs on the overall estimates. By systematically removing one SNP at a time and re-evaluating the estimates, researchers can identify whether a specific SNP has an outsized impact on the results. This step is essential for identifying potential sources of bias or undue influence in the analysis, contributing to the overall reliability and validity of the findings. Therefore, an indication of a causal association between exposure and outcome was judged to exist if the *p*-value was less than the Bonferroni-corrected significance level of 0.025 (*p*-value threshold = 0.05/2, accounting for 2 pairs of exposure and outcome). Statistical significance was defined as suggestive evidence of relationship with a *p*-value between 0.025.

R software version 4.1.1 and the “TwoSampleMR”, “MR-PRESSO”, and “MendelianRandomization” packages were used to carry out all MR statistical analysis and data visualization (Yavorska and Burgess, 2017; Hemani et al., 2018).

## Results

### Causal effect of circulating VEGF on IBD

**FIGURE 1:**
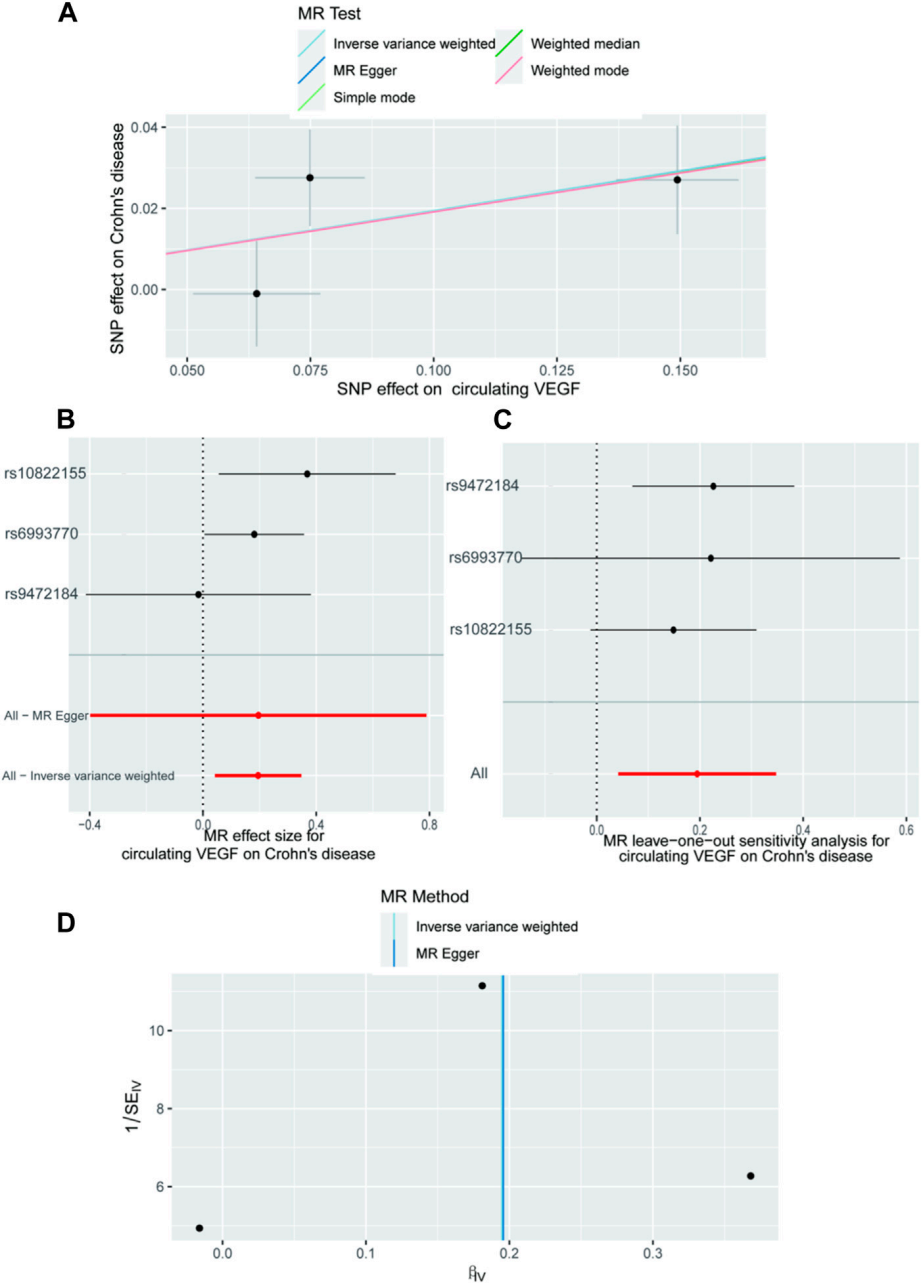
The causal impact of circulating VEGF on Crohn’s disease, according to the forward MR analysis. **(A)** Scatter plot showing the relationship between crohn’s disease and circulating VEGF. All four of the approaches used in the current manuscript were shown. **(B)** A forest plot was utilized to display the MR estimation and 95% CI values for each SNP. At the bottom are the MREgger and IVW findings. **(C)** Leave-one-out tests to determine if a single instrumental variable was responsible for the causal impact. **(D)** A funnel plot was used to determine if the connection that was seen was accompanied by noticeable heterogeneity. CI, confidence interval; IVW, inverse variance weighted; VEGF, Vascular Endothelial Growth Factor; MR, Mendelian randomization; SNPs, single‐nucleotide polymorphisms.

**FIGURE 2:**
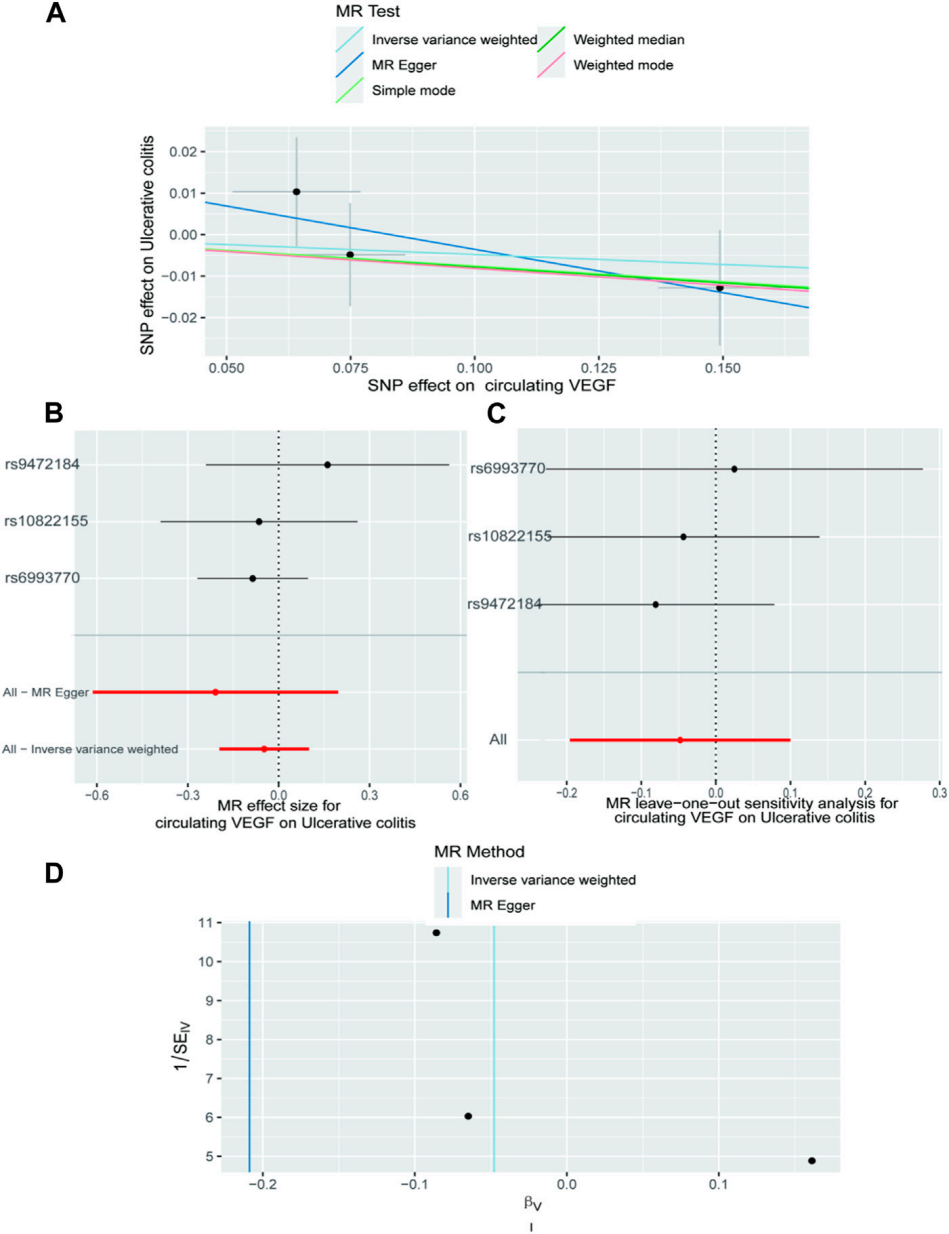
The causal impact of circulating VEGF on ulcerative colitis is examined using forward MR. The relationship between circulating VEGF and ulcerative colitis is depicted in a scatter plot in **(A)**. All four of the approaches used in the current manuscript were shown. **(B)** A forest plot was utilized to display the MR estimation and 95% CI values for each SNP. At the bottom are the MREgger and IVW findings. **(C)** Leave-one-out tests to determine if a single instrumental variable was responsible for the causal impact. **(D)** A funnel plot was used to determine if the connection that was seen was accompanied by noticeable heterogeneity. CI, confidence interval; IVW, inverse variance weighted; VEGF, Vascular Endothelial Growth Factor; MR, Mendelian randomization; SNPs, singleandhyphen;nucleotide polymorphisms.

In Supplementary Tables S2, S3, the three SNPs used as IVs for circulating VEGF are described in terms of their properties, along with the scatter plot in Figure 1. A strong causal connection between circulating VEGF and Crohn’s disease was noticed using the IVW MR approach (OR 1.21, 95% CI 1.04–1.42, *p* = 0.012). Additionally, a strong correlation between genetically predicted circulating VEGF and Crohn’s disease was found using the weighted median analysis (OR 1.21, 95% CI 1.03–1.43; *p* = 0.02) (Table 1). No significant associations were identified using other models. In contrast, the IVW investigation found no link between ulcerative colitis and circulating VEGF (OR 1.03, 95% CI 0.96–1.10; *p* = 0.395), which was consistent across other models. These findings are presented in Table 1 and Figure 2A (scatter plot), and Figure 2B (forest plots). No proof of possible directional pleiotropy was found, according to MR-Egger regression analysis (Table 2). Additionally, the funnel plot (Figure 2D) and the combined Cochran’s Q *p*-value in the IVW and MR-Egger techniques (Table 2, all *p*-values >0.001), a revealed no significant heterogeneity in the observed associations.

**TABLE 1:** Circulating VEGF and its association with IBD in the MR analyses.

Exposure	Outcome	No.of SNPs	Methods	OR (95% CI)	β (SE)	p
The forward MR analyses						
circulating VEGF	crohn’s disease	3	IVW	1.215 (1.043, 1.416)	0.195 (0.078)	0.013
			MR‐Egger	1.216 (0.672, 2.203)	0.196 (0.303)	0.635
			Weighted median	1.214 (1.033, 1.428)	0.194 (0.083)	0.019
			Weighted mode	1.211 (1.006, 1.458)	0.191 (0.095)	0.095
			Simple mode	1.211 (0.974, 1.506)	0.191 (0.111)	0.111
circulating VEGF	ulcerative colitis	4	IVW	0.953 (0.822, 1.105)	−0.048 (0.075)	0.526
			MR‐Egger	0.812 (0.541, 1.217)	−0.209 (0.207)	0.497
			Weighted median	0.925 (0.788, 1.087)	−0.078 (0.082)	0.344
			Weighted mode	0.922 (0.769, 1.104)	−0.082 (0.092)	0.470
			Simple mode	0.927 (0.750, 1.146)	−0.075 (0.108)	0.558
The reverse MR analyses						
crohn’s disease	circulating VEGF	121	IVW	1.009 (0.991, 1.028)	0.009 (0.009)	0.339
			MR‐Egger	0.985 (0.937, 1.036)	−0.015 (0.025)	0.566
			Weighted median	1.017 (0.988, 1.047)	0.017 (0.015)	0.257
			Weighted mode	1.015 (1.015, 1.068)	0.015 (0.026)	0.558
			Simple mode	1.015 (0.948, 1.087)	0.015 (0.035)	0.664
ulcerative colitis	circulating VEGF	86	IVW	1.016 (0.995, 1.038)	1.59e-02 (0.011)	0.140
			MR‐Egger	1.000 (0.950, 1.052)	1.44e-05 (0.026)	0.999
			Weighted median	1.012 (0.978, 1.048)	1.22e-02 (0.018)	0.486
			Weighted mode	1.025 (0.963, 1.090)	2.48e-02 (0.032)	0.435
			Simple mode	1.032 (0.958, 1.112)	3.20e-02 (0.038)	0.404

**TABLE 2:** Pleiotropy and heterogeneity analyses.

Exposure	Outcome	No. of SNPs	MR‐Egger regression		MR PRESSO		Heterogeneity analyses		
			Intercept	p_intercept	Global test p	Correct p^a^	Method	Q	Q_pval
circulating VEGF	crohn’s disease	3	−0.000	0.998	-	-	IVW	2.291	0.318
							MR Egger	2.291	0.130
circulating VEGF	ulcerative colitis	3	0.017	0.557	-	-	IVW	1.219	0.471
							MR Egger	0.520	0.544
crohn’s disease	circulating VEGF	121	0.003	0.318	148.941	0.369	IVW	138.992	0.113
							MR Egger	137.829	0.114
ulcerative colitis	circulating VEGF	86	0.002	0.504	86.361	0.484	IVW	79.282	0.654
							MR Egger	78.831	0.639

Abbreviations: IVW, inverse variance weighted; MR PRESSO, mendelian randomization pleiotropy residual sum and outlier; VEGF, vascular endothelial growth factor; SNPs, single‐nucleotide polymorphisms.

^a^ Correct p is computed by eliminating instrument variations with horizontal pleiotropy if the MR PRESSO, global test finds the pleiotropy and there is a substantial difference between the results before and after the outlier is removed.

### Causal effect of IBD on circulating VEGF

**FIGURE 3:**
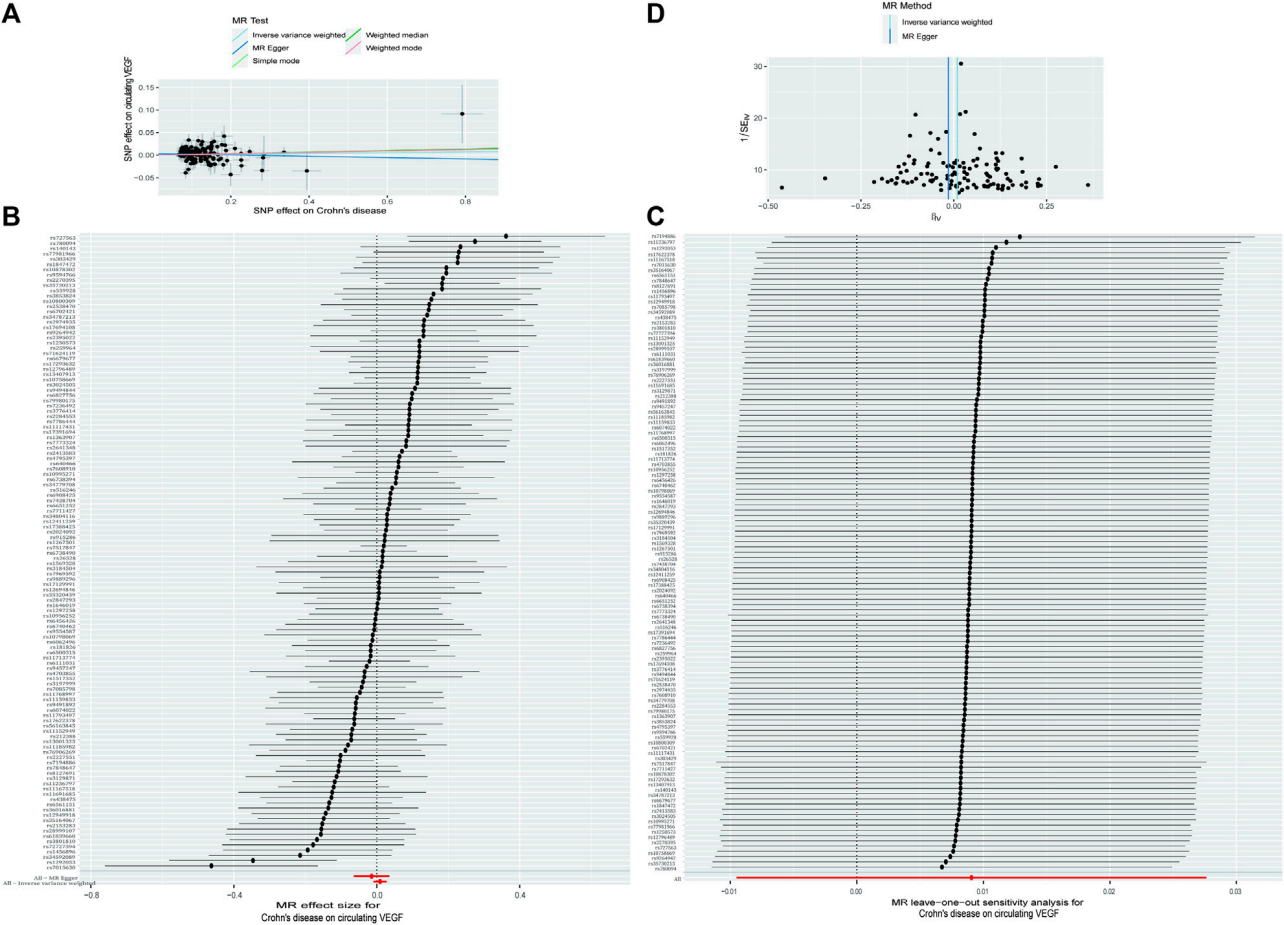
The causal impact of Crohn’s disease on circulating VEGF was examined using reverse MR. **(A)** A scatter plot showing the relationship between circulating VEGF and crohn’s disease. The current book includes illustrations for each of the four ways. **(B)** The MR estimate and 95% CI values (the black line segment) for each SNP are shown on a forest plot, along with the MR-Egger MR and IVW findings at the bottom. **(C)** Leave-one-out analyses were used to see if any particular instrumental variable was responsible for the causal impact. Applying the funnel plot to examine the observed correlation for any obvious heterogeneity **(D)**. CI, confidence interval; IVW, inverse variance weighted; VEGF, Vascular Endothelial Growth Factor; MR, Mendelian randomization; SNPs, singleandhyphen;nucleotide polymorphisms.

**FIGURE 4:**
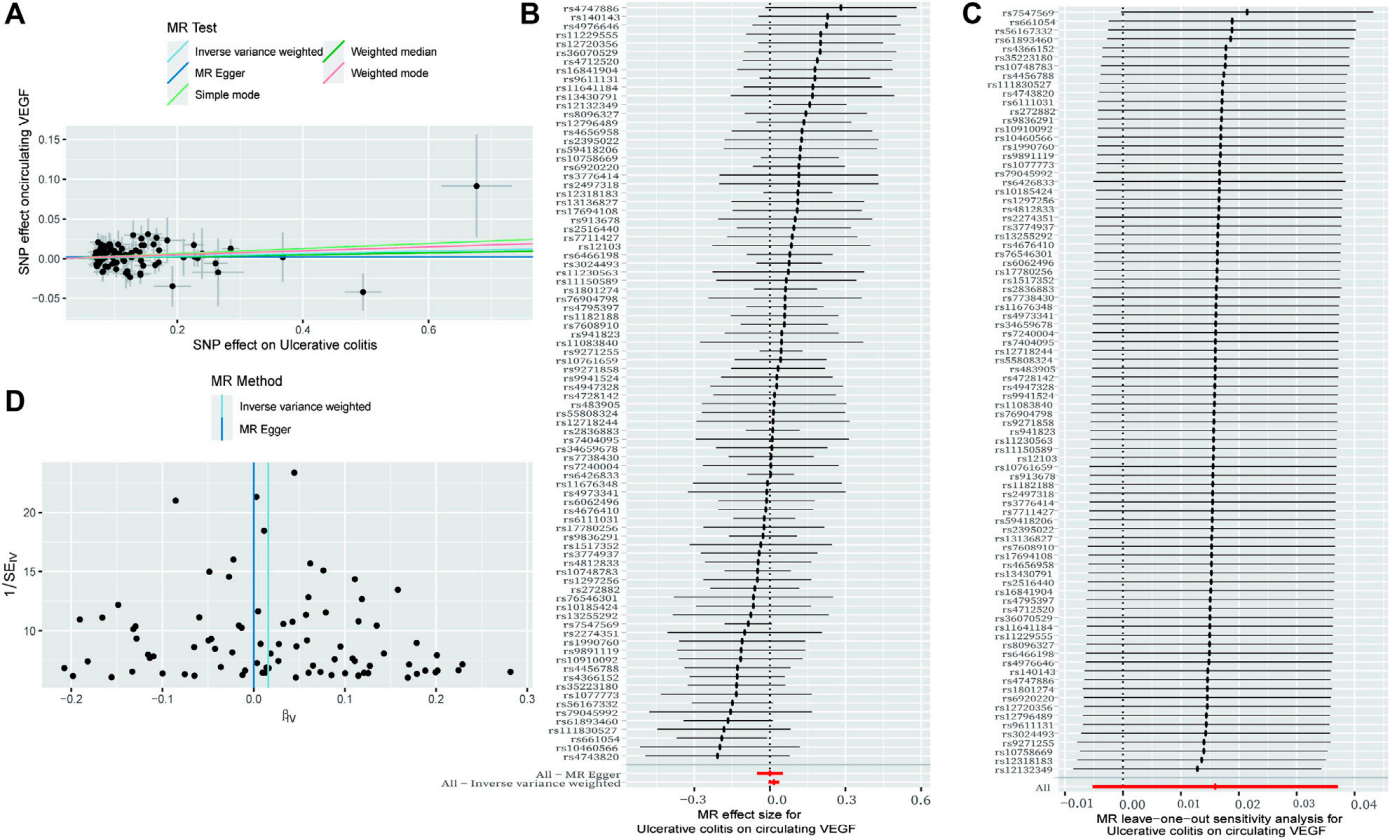
The causal impact of Crohn’s disease on circulating VEGF was examined using reverse MR. **(A)** A scatter plot showing the relationship between circulating VEGF and ulcerative colitis. The current book includes illustrations for each of the four ways. **(B)** The MR estimate and 95% CI values (the black line segment) for each SNP are shown on a forest plot, along with the MR-Egger MR and IVW findings at the bottom. **(C)** Leave-one-out analyses were used to see if any particular instrumental variable was responsible for the causal impact. Applying the funnel plot to examine the observed correlation for any obvious heterogeneity **(D)**. CI, confidence interval; IVW, inverse variance weighted; VEGF, Vascular Endothelial Growth Factor; MR, Mendelian randomization; SNPs, singleandhyphen;nucleotide polymorphisms.

As demonstrated in Table 1 and Figure 3A (scatter plot), Figure 3B (forest plot), Figure 4A (scatter plot), and Figure 4B (forest plot), no causal connection was found by the IVW analysis between Crohn’s disease (121 SNPs as IVs, Supplementary Table S4) or ulcerative colitis (86 SNPs as IVs, Supplementary Table S5) and circulating VEGF (OR 1.01, 95% CI 0.99–1.03; *p* = 0.339 and OR 1.02, 95% CI 0.99–1.04; *p* = 0.140, respectively). The last three models yielded results consistent with the IVW analysis (Table 1). Both MR-Egger regression analyses (Table 2, intercept = 0.003, *p* = 0.318 and intercept = 0.002, *p* = 0.504) and the MR-PRESSO global test (Table 2, *p* = 0.369 and *p* = 0.484) lacked any indication of possible pleiotropy. Cochran’s Q test using the IVW method (Table 2, Q = 138.992, *p* = 0.113 and Q = 79.282, *p* = 0.654) and the MR-Egger method (Table 2, Q = 137.829, *p* = 0.114 and Q = 78.831, *p* = 0.639) suggested that the IVs were not heterogeneous. Additionally, the leave-one-out analyses (Figures 3C, 4C) indicated that the observed associations remained stable, even after the removal of any single SNP.

## Discussion

In our bidirectional two-sample Mendelian randomization (MR) investigation, we made intriguing findings regarding the relationship between genetically proxied high levels of circulating VEGF and inflammatory bowel disease (IBD). While we established a significant link between elevated VEGF and Crohn’s disease, we did not find a strong association with ulcerative colitis. Moreover, our analysis indicated that neither Crohn’s disease nor ulcerative colitis had a causative role in driving higher levels of circulating VEGF. These insights contribute to a deeper understanding of the complex interplay between genetic factors, VEGF, and the distinct manifestations of IBD.

As previously noted, there is a body of observational evidence linking circulating vascular endothelial growth factor (VEGF) to inflammatory bowel disease (IBD) (Koutroubakis et al., 2006). However, when examining specific functional VEGF single nucleotide polymorphisms (SNPs) such as YC2578A, YG1154A, YG634C, and C936T, one study found that these SNPs were not strongly associated with IBD susceptibility in a large research population. Furthermore, this study did not identify consistent differences in circulating soluble VEGF (sVEGF) levels based on VEGF genotypes and haplotypes. While it was established that IBD patients exhibited higher sVEGF levels, the variations were not substantial when compared to individuals with gastrointestinal (GI) illnesses other than IBD. Interestingly, in a cohort study, it was observed that only Crohn’s disease patients with specific genotypes, such as -G1154A and −2578/-1154/-634 AAG promoter haplotype, had sVEGF levels affected by VEGF polymorphisms (Ferrante et al., 2006). These nuanced findings highlight the complex interplay between VEGF polymorphisms and the distinct manifestations of IBD, shedding light on the variability in responses across different subtypes of the disease. Our findings support the hypothesis that increased sVEGF levels contribute to susceptibility to Crohn’s disease. In combination with the results of the cohort study by Marc Ferrante et al., we concluded that the VEGF polymorphisms investigated do not influence IBD susceptibility or predict sVEGF levels. Nevertheless, several potential mechanisms underlying this causal relationship should be considered. While increased sVEGF levels may not be a defining trait of IBD, they may be a byproduct of active inflammation, Crohn’s disease is a form of granulomatous enteritis frequently accompanied by inflammatory hyperplasia and intestinal strictures. Emerging research has provided intriguing insights into the potential mechanisms underlying the elevated levels of vascular endothelial growth factor (VEGF) in specific disease contexts. For instance, one study demonstrated that serosal fibroblasts within Crohn’s disease strictures exhibit an enhanced capacity to produce VEGF (Beddy et al., 2004). Additionally, another investigation uncovered that multinuclear giant cells in lung sarcoid granulomas are a source of VEGF production (Morohashi et al., 2003). These findings suggest a possible link between the VEGF single nucleotide polymorphisms (SNPs) selected as instrumental variables (IVs) in our study and the development of Crohn’s disease characterized by granulomatous enteritis. This highlights the intricate relationship between genetic factors, VEGF regulation, and the distinct pathophysiological features of Crohn’s disease, offering valuable avenues for further research and potential therapeutic targets. However, since the number of SNPS is valid, the results of this study still need to be further verified.

In conclusion, our study harnessed the power of comprehensive genetic summary data to delve into the intricate relationship between circulating vascular endothelial growth factor (VEGF) and inflammatory bowel disease (IBD). The results of our investigation shed light on a unidirectional causal effect, revealing that elevated levels of circulating VEGF significantly contribute to the susceptibility to Crohn’s disease. This unidirectional link underscores the importance of VEGF in the pathogenesis of Crohn’s disease and opens avenues for further research aimed at better understanding the molecular mechanisms at play. These findings carry significant implications for the development of novel therapeutic strategies, targeting VEGF pathways in the management of Crohn’s disease and potentially other related conditions.

## Data Availability

The original contributions presented in the study are included in the article/Supplementary Material, further inquiries can be directed to the corresponding author.
