# Supplementary material for: Circulating VEGF and inflammatory bowel disease: a bidirectional mendelian randomization
Source: Front Genet. 2024 Apr 18;15:1282471. doi: 10.3389/fgene.2024.1282471 (PMC11063361; doi:10.3389/fgene.2024.1282471)
Supplement: Supplementary file 1 [file Table1.DOCX]

| **Supplemetary Table 1.** Details of VEGF data sources used in this study. | | | | | | | | | | | | | |
| --- | --- | --- | --- | --- | --- | --- | --- | --- | --- | --- | --- | --- | --- |
| **Study name** | **IMPROVE** | **STANLEY** | **EpiHealth** | **PIVUS** | **ULSAM** | **INTERVAL** | **LifeLines-DEEP** | **NSPHS** | **STABILITY** | **Estonian BB** | **ORCADES** | **VIS** | **MPP-RES** |
| **Study name, long** | Carotid Intima Media Thickness [IMT] and IMT-Progression as Predictors of Vascular Events in a High Risk European Population study | STANLEY / SWEBIC | Epidemiology for Health | The Prospective Investigation of the Vasculature in Uppsala Seniors | The Uppsala Longitudinal Study of Adult Men | The INTERVAL study | The Prospective Cohort Study | The Northern Sweden Population Health Study | the Stabilization of Atherosclerotic Plaque by Initiation of Darapladib Therapy trial | Estonian Genome Center, Institute of Genomics, University of Tartu | The Orkney Complex Disease Study | Vis | The Malmö Preventive Project |
| **PI name** | Anders Hamsten, Elena Tremoli | Mikael Landen | Lars Lind | Lars Lind | Lars Lind | John Danesh, Adam Butterworth | Jingyuan Fu, Lude Franke | Ulf Gyllensten | Lars Wallentin, Angeta Siegbahn | Tõnu Esko | Jim Wilson | Caroline Hayward | J. Gustav Smith |
| **PI e-mail** | [anders.hamsten@ki.se](mailto:anders.hamsten@ki.se) | [mikael.landen@gu.se](mailto:mikael.landen@gu.se) | [lars.lind@medsci.uu.se](mailto:lars.lind@medsci.uu.se) | [lars.lind@medsci.uu.se](mailto:lars.lind@medsci.uu.se) | [lars.lind@medsci.uu.se](mailto:lars.lind@medsci.uu.se) | [asb38@medschl.cam.ac.uk](mailto:asb38@medschl.cam.ac.uk) | [fjingyuan@gmail.com](mailto:fjingyuan@gmail.com) | [ulf.gyllensten@igp.uu.se](mailto:ulf.gyllensten@igp.uu.se) | lars.wallentin@ucr.uu.se, agneta.siegbahn@medsci.uu.se | [tesko@broadinstitute.org](mailto:tesko@broadinstitute.org) | [jim.wilson@ed.ac.uk](mailto:jim.wilson@ed.ac.uk) | Caroline.Hayward@igmm.ed.ac.uk | gustav.smith@med.lu.se |
| **Analyst name** | Anders Malarstig |  | Stefan Gustafsson | Stefan Gustafsson | Stefan Gustafsson | James Peters | Daria Zhernakova | Stefan Enroth | Niclas Eriksson | Anette Kalnapenkis | Andrew Bretherick | Thibaud Boutin | J. Gustav Smith |
| **Analyst e-mail** | [anders.malarstig@ki.se](mailto:anders.malarstig@ki.se) | [anders.malarstig@ki.se](mailto:anders.malarstig@ki.se) | [stefan.gustafsson@medsci.uu.se](mailto:stefan.gustafsson@medsci.uu.se) | [stefan.gustafsson@medsci.uu.se](mailto:stefan.gustafsson@medsci.uu.se) | [stefan.gustafsson@medsci.uu.se](mailto:stefan.gustafsson@medsci.uu.se) | [jp549@medschl.cam.ac.uk](mailto:jp549@medschl.cam.ac.uk) | [dasha.zhernakova@gmail.com](mailto:dasha.zhernakova@gmail.com) | [stefan.enroth@igp.uu.se](mailto:stefan.enroth@igp.uu.se) | niclas.eriksson@ucr.uu.se | [anette.kalnapenkis@ut.ee](mailto:anette.kalnapenkis@ut.ee) | [a.bretherick@ed.ac.uk](mailto:a.bretherick@ed.ac.uk) | [Thibaud.Boutin@igmm.ed.ac.uk](mailto:Thibaud.Boutin@igmm.ed.ac.uk) | gustav.smith@med.lu.se |
| **Reference(s)** | PMID: 23152477 |  | PMID: 23435790 | PMID: 16141402 | PMID: 16030278 | PMID: 27863252 | PMID: 26319774 | PMID:19060911 & 20568910 | PMID: 24678955 | PMID:24518929 | PMID: 18760389 |  | PMID: 20211303 |
| **Design** | Patients with metabolic syndrome | Bipolar cases and controls | Population-based | Population-based | Population-based | RCT (blood donors) | Population-based | Population-based | RCT | Population-based | Population-based isolate | Population-based isolate | Population-based, oversampling of subjects with diabetes and impaired fasting glucose |
| **Ethnicity** | European | Swedish | European | European | European | European | European | European | Worldwide (78% reported with ethnic group white in main trial) | European | European | European | European |
| **Sample size, total** | 3,700 | 10,000 | 2,500 | 1,016 | 1,221 | 4,994 | 1539 | 1,067 | 15828 | 500 | 2,078 | 960 | 1792 |
| **GWAS, call rate** |  | 98% | ≥ 98% | ≥ 95% | ≥ 95% | Sample call rate >=98%. | ≥ 95% | ≥ 95% | ≥ 95% | ≥ 95% | >=98% | >95% | Sample call rate >=98%. |
| **GWAS, other exclusions** | 1) Gender mismatch 2) Heterozygosity 3) Cryptic relatedness. | 1) Gender mismatch; 2) Heterozygosity 3) Cryptic relatedness. | 1) Gender mismatch; 2) Ethnic outliers; 3) Heterozygosity > 5 s.d. from the mean; 4) Cryptic relatedness. | 1) Gender mismatch; 2) Ethnic outliers; 3) Heterozygosity > 5 s.d. from the mean; 4) Cryptic relatedness. | 1) Gender mismatch; 2) Ethnic outliers; 3) Heterozygosity > 5 s.d. from the mean; 4) Cryptic relatedness. | 1) Sex mismatches 2) low call rates 3) duplicate samples 4) heterozygosity >3 sd from mean 5) non-European ancestry 6) relatedness (pi_hat> 0.187) | 1) relavtives; 2) Ethnic outliers; 3) sample mismatch | 1) Gender mismatch; 2) Heterozygosity; 3) Genetic outliers through multidimentional scaling | 1) Gender mismatch 2) Heterozygozity checked but none excluded | 1) Gender mismatch; 2) Ethnic outliers; 3) Heterozygosity > 3 s.d. from the mean; 4) Cryptic relatedness. | Ethnic outliers; duplicates; gender mismatch; excess IBS incompatible with pedigree |  | 1) Gender mismatch; 2) Ethnic outliers; 3) Heterozygosity within +/-3 sd from the mean; 4) Genetic outliers through multidimentional scaling plots |
| **OLINK, exclusions** | 1) Technical failures; 2) None | 1) Technical failures; 2) None | 1) Technical failures; 2) Missing > 5% proteins on CVDII or CVDIII. | 1) Technical failures; 2) Missing > 5% proteins on CVDI. | 1) Technical failures; 2) Missing > 2% proteins on CVDI. | "Flagged" samples (technical failures) | 1) Technical failures; 2) None | 1) Technical failures; 2) None | 1) Technical failures | 1) Technical failures | 1) Technical failures; 2) None |  | 1) Technical failures; 2) None. |
| **Sample size, SCALLOP (max)** | 3,403 | 681 | 2,335 | 933 | 730 | 4,987 (post QC) | 1178 | 965 | 2,967 | 496 (post QC) | 971 |  | 882 |
| **OLINK panel(s)** | CVD I | CVD I, INF I, ONC I | CVDII, CVDIII | CVDI | CVDI | INF, CVD2, CVD3 | CVDIII | ONC I, CVD I | CVD-I, INF-I | CVD2, CVD3, INF, ONC II | CVDII, CVDIII, INFI, cardiometabolic, metabolic, immune response, neurology, neuro exploratory, oncology II, cell regulation, development, organ damage | CVDII, CVDIII, INFI | CVDIII |
| **Pre-imputation QC** |  |  |  |  |  |  |  |  |  |  |  |  |  |
| **Genotyping array** | Cardiometabochip, Immunochip | OmniExpress and PsychChip | Illumina HumanCoreExome | Illumina OmniExpress+Metabochip | Illumina Omni2.5+Metabochip | Affymetrix Axiom UK Biobank array | HumanCytoSNP-12 BeadChip, ImmunoChip | llumina OmniExpress or HapMap300v2 + Human Exome | Illumina HumanOmniExpressExome-8 v1 | Illumina HumanOmniExpress | Illumina HumanHap300v1, Omni1, OmniX | Illumina HumanHap300v1 | Infinium PsychArray-24 v1.2 |
| **Genotype calling algorithm** |  | Birdsuite, z-call | GenCall + zCall (Oxford protocol) | GenCall | GenCall | Affymetrix Power Tools | NA | GenomeStudio(v2011.1) + zCall | Genomestudio + zCall | Illumina Genomestudio |  | Beadstudio-Gencall v3.0 | GenomeStudio(v1.6.2.2) + Birdseed v1.33 (concordant calls kept), zCall (maf <1%) |
| **MAF inclusion cutoff** | ≥ 0.5% | ≥ 1% | ≥ 0.5% | ≥ 1% | ≥ 1% | All results provided without MAF filter (although filter used for imputation scaffold) | > 5% | ≥ 1% | ≥ 0.1% | ≥ 1% | ≥0.01 | ≥0.01 | >0.5 |
| **Call rate inclusion cutoff** | ≥ 98 % | ≥ 98 % | ≥ 97% (GenCall), ≥ 99% (zCall) | ≥ 99% (MAF < 5%) or ≥ 95% (MAF ≥ 5%) | ≥ 99% (MAF < 5%) or ≥ 95% (MAF ≥ 5%) | Samples called in 10 batches, within-batch variant call rate inclusion > 0.97. See Astle et al Cell 2016 for details of QC post batch merging. Variants were dropped from all batches if they failed in at least four of the batches due to deviation from HWE, low call rate or Affymetrix variant exclusion criteria. a call rate filter of 99% over the batches that a variant was not failed in, and a global call rate filter of 75% (effectively ensuring a variant passed in at least eight of the ten batches). | >95% | > 95% SNP, > 90% Individual | >95% | ≥ 98 % | 97% | 98% | >95% sample, >97% snp |
| **HWE p-value inclusion cutoff** | ≥ 10-8 | 1e-10 for cases, 1e-6 for controls | ≥ 10-4 | > 10-6 | > 10-6 | 5x10^-6 | > 10-4 | > 3.2/1.4 x 10-8 | none applied | > 10-6 | 1.00E-06 | 1.00E-06 | > 10-6 |
| **Imputation** |  |  |  |  |  |  |  |  |  |  |  |  |  |
| **Phasing** | NA | Shapeit | SHAPEIT2, v2.r727 | NA | NA | SHAPEIT3 | shapeit2 | SHAPEIT version 2.5 (r790) | SHAPEIT v2 (r837) | Eagle 2.3 | SHAPEIT version 2.5 (r790) + duoHMM | SHAPEIT version 2.5 (r790) + duoHMM | Eagle v2.3 |
| **Imputation software** | MiniMac | Impute2 | IMPUTE2, v.2.3.0 | IMPUTE2, v.2.2.2 | IMPUTE2, v.2.2.2 | Sanger imputation server (PBWT imputation algorithm) | Minimac3 (Michigan Imputation Server) | IMPUTE2 (v2.3.2) | IMPUTE2 (v2.3.1) | Beagle 2.4.2 | Sanger imputation server (PBWT imputation algorithm) | Sanger imputation server (PBWT imputation algorithm) | Minimac3 (Michigan Imputation Server v 1.0.1) |
| **Imputation reference panel** | 1000G, phase 1 (v5) | 1000G | 1000G, phase 1 (v3) | 1000G, phase 1 (v3) | 1000G, phase 1 (v3) | Combined 1000G phase 3 +UK10K reference panel | HRC | 1000G Phase 3 | 1000G Phase 3 | 1000G Phase 3 | HRC.r1-1 | HRC.r1-1 | HRC r1.1 |
| **Below LOD imputation** | LOD/2 | No | No | Yes (LOD/2 replace) | Yes (LOD/2 replace) | No- non-truncated values used |  | No | Yes (LOD/2) | Yes (LOD/2) | Yes (replace with 0) | No | No |
| **Trait transformation** | Rank-based inverse normal transformation (Blom) | Rank-based inverse normal transformation (Blom) | Rank-based inverse normal transformation (Blom) | Rank-based inverse normal transformation (Blom) | Rank-based inverse normal transformation (Blom) | Rank-based inverse normal transformation | z-score transformation | Rank-based inverse normal transformation | Rank-based inverse normal transformation | Rank-based inverse normal transformation | Rank-based inverse normal transformation | Rank-based inverse normal transformation | z-score transformation |
| **Software** | Plink 1.9 | Plink 1.9 | SNPTEST v.2.5.2 | SNPTEST v.2.5.2 | SNPTEST v.2.5.2 | SNPTEST v2.5.2 | https://github.com/molgenis/systemsgenetics/tree/master/eqtl-mapping-pipeline | ProbABEL/palinear (v1.8.0) | SNPTEST v2.5.2 | Plink 1.9 | GenABEL+RegScan | GenABEL+RegScan | SNPTEST v.2.5.2 |
| **Software, method** | dosage | dosage | -frequentist 1 -method 'em' | -frequentist 1 -method 'em' | -frequentist 1 -method 'em' | -method expected | Spearman's correlation | dosage | -frequentist 1 -method score | dosage | LMM (Grammar Gamma) + genome scan | LMM (Grammar Gamma) + genome scan | -frequentist 1 -method expected |
| **Adjustment** | site, age, sex, olink_batch | age, sex, MDS components, olink batch | Age, sex, OLINK plate, MDS component 1-5 | Age, sex, OLINK plate, storage time, MDS component 1-2 | Age, OLINK plate, storage time, MDS component 1-2 | Protein abundance was regressed on age, sex, season, plate, bleed to processing time (days), before IVNT. PLINK MDS 1-3 used as covariates in SNPTEST | age, gender, smoking status, oral contraceptive usage, blood cell counts | selection of 159 covariates per protein, also including storage time. (http://www.tandfonline.com/doi/full/10.3109/1354750X.2015.1093546) | AGE, SEX, PC1, PC2, PC3, PC4 | age, sex, OLINK plate, MDS components 1-10 | age+sex+array+time_in_storage_days+season_of_vene+plate_no+plate_row+plate_column+pc{1:10} | Age+sex | Age, sex, Olink plate |
| **Info metric from** | MiniMac, Plink INFO | PLINK INFO | SNPTEST v.2.5.2 | SNPTEST v.2.5.2 | SNPTEST v.2.5.2 | SNPTEST v2.5.2 | minimac3 | IMPUTE2's 'info'-score. | IMPUTE2's 'info'-score from SNPTEST. | Plink INFO | IMPUTE2 INFO score | IMPUTE2 INFO score | minimac3 |

| **Supplementary Table 2.** Summary of genetic variants (n=3) used to estimate the effect of circulating VEGF on crohn’s disease in MR analyses. | | | | | | | |
| --- | --- | --- | --- | --- | --- | --- | --- |
| **rsid** | **Chr** | **Pos** | **Effect allele** | **Other allele** | **P-value** | **Beta** | **Se** |
| rs10822155 | 10 | 65071215 | A | C | 0.069751321 | 0.1487520 | 0.08202380 |
| rs6993770 | 8 | 106581528 | T | A | 0.236082147 | 0.2212725 | 0.18675365 |
| rs9472184 | 6 | 43944859 | A | G | 0.004698502 | 0.2260790 | 0.07997111 |

| **Supplementary Table 3.** Summary of genetic variants (n=3) used to estimate the effect of circulating VEGF on ulcerative colitis in MR analyses. | | | | | | | |
| --- | --- | --- | --- | --- | --- | --- | --- |
| **rsid** | **Chr** | **Pos** | **Effect allele** | **Other allele** | **P-value** | **Beta** | **Se** |
| rs6993770 | 8 | 106581528 | T | A | 0.8475019 | 0.02478796 | 0.12889804 |
| rs10822155 | 10 | 65071215 | A | C | 0.6404500 | -0.04346806 | 0.09306545 |
| rs9472184 | 6 | 43944859 | A | G | 0.3198683 | -0.08074716 | 0.08117507 |

| **Supplementary Table** 4. Summary of genetic variants (n=121) used to estimate the effect of crohn’s disease on circulating VEGF in MR analyses. | | | | | | | |
| --- | --- | --- | --- | --- | --- | --- | --- |
| **rsid** | **Chr** | **Pos** | **Effect allele** | **Other allele** | **P-value** | **Beta** | **Se** |
| rs10758669 | 9 | 4981602 | A | C | 0.132472028 | 0.113522538 | 0.075459098 |
| rs10798069 | 1 | 186875459 | T | G | 0.941492198 | -0.011365735 | 0.154858141 |
| rs10800309 | 1 | 161472158 | G | A | 0.23013934 | 0.152640346 | 0.127200288 |
| rs10878302 | 12 | 40669826 | A | T | 0.140635139 | 0.194552529 | 0.132040569 |
| rs10956252 | 8 | 126536137 | G | C | 0.978819712 | -0.002528637 | 0.09524532 |
| rs10995271 | 10 | 64438486 | C | G | 0.344176157 | 0.055954341 | 0.059151732 |
| rs11117431 | 16 | 86015316 | G | A | 0.328184244 | 0.088430361 | 0.090440142 |
| rs11152949 | 6 | 106449085 | G | A | 0.439457731 | -0.068734171 | 0.088906155 |
| rs11159833 | 14 | 88476004 | T | C | 0.652150134 | -0.056046951 | 0.12433404 |
| rs11167518 | 5 | 150258920 | A | C | 0.126162398 | -0.120832097 | 0.079005602 |
| rs11185982 | 10 | 82305627 | C | T | 0.567709166 | -0.080631122 | 0.141104463 |
| rs11236797 | 11 | 76299649 | A | C | 0.050686029 | -0.1178508 | 0.060308625 |
| rs11691685 | 2 | 145481827 | G | A | 0.350484831 | -0.124931034 | 0.133809382 |
| rs11713774 | 3 | 18765978 | C | T | 0.885697887 | -0.017337293 | 0.120607258 |
| rs11768997 | 7 | 50533716 | T | G | 0.688604884 | -0.047276937 | 0.117971422 |
| rs11793497 | 9 | 139271850 | G | A | 0.393959377 | -0.061566877 | 0.072222683 |
| rs12411259 | 1 | 172866210 | A | G | 0.78606143 | 0.028281385 | 0.104194576 |
| rs1250573 | 10 | 81042475 | A | G | 0.162505545 | 0.119271947 | 0.085395891 |
| rs1267501 | 6 | 14715257 | C | T | 0.894298461 | 0.021794314 | 0.164030893 |
| rs12694846 | 2 | 231148128 | G | A | 0.955341807 | 0.006067646 | 0.108350814 |
| rs12796489 | 11 | 3059360 | A | C | 0.158849771 | 0.115311265 | 0.081841949 |
| rs1292053 | 17 | 57963537 | G | A | 0.003742563 | -0.346546706 | 0.11953668 |
| rs12949918 | 17 | 40526273 | C | T | 0.175562422 | -0.143025812 | 0.105589526 |
| rs1297258 | 21 | 16806709 | T | C | 0.992876125 | 0.000785879 | 0.088018484 |
| rs13001325 | 2 | 102939036 | T | C | 0.444142105 | -0.071727241 | 0.093734462 |
| rs13407913 | 2 | 25097644 | G | A | 0.242144723 | 0.114402487 | 0.09780976 |
| rs1363907 | 5 | 96252803 | A | G | 0.413253375 | 0.087740678 | 0.107238606 |
| rs140143 | 22 | 30173109 | T | G | 0.100821502 | 0.233959589 | 0.142581433 |
| rs1456896 | 7 | 50304461 | T | C | 0.10922309 | -0.193433298 | 0.120767879 |
| rs1517352 | 2 | 191931464 | C | A | 0.802587349 | -0.034995757 | 0.139983027 |
| rs1569328 | 14 | 75741751 | T | C | 0.9079329 | 0.015565627 | 0.134596896 |
| rs1646019 | 16 | 11359680 | T | C | 0.986361612 | 0.001795671 | 0.105046732 |
| rs17129991 | 1 | 67862986 | T | C | 0.964724222 | 0.006327757 | 0.14307761 |
| rs17293632 | 15 | 67442596 | T | C | 0.244398684 | 0.116058076 | 0.099700897 |
| rs17388425 | 5 | 158824174 | G | C | 0.770752505 | 0.027980388 | 0.096023605 |
| rs17391694 | 1 | 78623626 | T | C | 0.550780542 | 0.088197495 | 0.147835801 |
| rs17622378 | 5 | 131778452 | G | A | 0.279661048 | -0.063092478 | 0.058360542 |
| rs17694108 | 19 | 33731551 | A | G | 0.405408945 | 0.130670503 | 0.157055893 |
| rs181826 | 5 | 141526057 | A | C | 0.87935595 | -0.017069164 | 0.11245567 |
| rs1847472 | 6 | 90973159 | A | C | 0.096153783 | 0.226033959 | 0.135854607 |
| rs2024092 | 19 | 1124031 | A | G | 0.770051378 | 0.025734099 | 0.088037707 |
| rs212388 | 6 | 159490436 | T | C | 0.506923078 | -0.071305078 | 0.107446008 |
| rs2153283 | 10 | 59972299 | A | C | 0.19815644 | -0.15256792 | 0.118561818 |
| rs2227551 | 10 | 75669190 | T | G | 0.399974561 | -0.101563066 | 0.120668989 |
| rs2270395 | 16 | 50846832 | T | C | 0.074595137 | 0.184994531 | 0.103757802 |
| rs2284553 | 21 | 34776695 | G | A | 0.413704843 | 0.091092333 | 0.111442747 |
| rs2395022 | 7 | 98750379 | C | A | 0.419267733 | 0.130396496 | 0.16144328 |
| rs2413583 | 22 | 39659773 | T | C | 0.320569336 | 0.070339865 | 0.070815134 |
| rs2538470 | 7 | 148220448 | G | A | 0.342989036 | 0.146736513 | 0.154740323 |
| rs259964 | 20 | 57824309 | G | A | 0.443815687 | 0.119157431 | 0.155605586 |
| rs2641348 | 1 | 120437884 | G | A | 0.569379541 | 0.081591613 | 0.143403442 |
| rs26528 | 16 | 28517709 | C | T | 0.865291016 | 0.015853811 | 0.093454045 |
| rs2847293 | 18 | 12782448 | T | A | 0.95150988 | 0.005392095 | 0.08867001 |
| rs28999107 | 12 | 6493100 | T | G | 0.247467813 | -0.155266089 | 0.134252633 |
| rs2974935 | 1 | 155181843 | T | G | 0.358916734 | 0.132361447 | 0.144273978 |
| rs3024505 | 1 | 206939904 | A | G | 0.212519746 | 0.113120119 | 0.090738063 |
| rs303429 | 10 | 30708441 | T | C | 0.119100898 | 0.226809098 | 0.145524912 |
| rs3129871 | 6 | 32406342 | C | A | 0.385163874 | -0.112599804 | 0.129660381 |
| rs3184504 | 12 | 111884608 | C | T | 0.940707891 | 0.013303573 | 0.178859145 |
| rs3197999 | 3 | 49721532 | A | G | 0.611120564 | -0.038681228 | 0.076073082 |
| rs34592089 | 4 | 102926923 | A | G | 0.099732089 | -0.214637473 | 0.13038725 |
| rs34779708 | 10 | 35466185 | G | T | 0.533411346 | 0.052804986 | 0.08478547 |
| rs34787213 | 11 | 60799046 | T | C | 0.192756062 | 0.140920323 | 0.108194751 |
| rs34804116 | 5 | 72539850 | A | C | 0.809499757 | 0.02875136 | 0.119264902 |
| rs35164067 | 19 | 10525181 | A | G | 0.114616671 | -0.148563537 | 0.094159988 |
| rs35320439 | 2 | 242737341 | C | T | 0.967574599 | 0.005948451 | 0.146331888 |
| rs35730213 | 1 | 200874229 | C | G | 0.026027148 | 0.182654446 | 0.082062142 |
| rs36016881 | 1 | 8051241 | G | A | 0.290446394 | -0.135828416 | 0.128486339 |
| rs3776414 | 5 | 10689562 | G | T | 0.473488774 | 0.09117997 | 0.127201687 |
| rs3801810 | 7 | 26892531 | A | G | 0.179106421 | -0.167493029 | 0.124668107 |
| rs3853824 | 17 | 54880993 | C | T | 0.261973374 | 0.158552234 | 0.141345015 |
| rs438475 | 6 | 32186245 | A | G | 0.212429187 | -0.126983329 | 0.101838115 |
| rs4703855 | 5 | 71693899 | T | C | 0.834968706 | -0.034124568 | 0.163797928 |
| rs4795397 | 17 | 38023745 | G | A | 0.440919039 | 0.063473832 | 0.082364854 |
| rs516246 | 19 | 49206172 | T | C | 0.661748776 | 0.042766373 | 0.097751711 |
| rs559928 | 11 | 64150370 | C | T | 0.196060472 | 0.182599572 | 0.141237238 |
| rs56163845 | 5 | 173373948 | G | A | 0.617075077 | -0.063106585 | 0.12621317 |
| rs6062496 | 20 | 62329099 | A | G | 0.892505031 | -0.01249188 | 0.092439914 |
| rs6074022 | 20 | 44740196 | T | C | 0.64574778 | -0.059177065 | 0.128736071 |
| rs6111031 | 20 | 1682037 | T | C | 0.721969213 | -0.020540717 | 0.057726497 |
| rs61839660 | 10 | 6094697 | T | C | 0.248406355 | -0.15658853 | 0.135665063 |
| rs640466 | 19 | 34670725 | C | T | 0.689156517 | 0.060678651 | 0.151696628 |
| rs6456426 | 6 | 21438889 | A | C | 0.970992409 | -0.004033092 | 0.110910017 |
| rs6500315 | 16 | 50508101 | G | A | 0.854151897 | -0.017177526 | 0.093445743 |
| rs6561151 | 13 | 44484706 | A | G | 0.137583965 | -0.133199228 | 0.089705603 |
| rs6651252 | 8 | 129567181 | C | T | 0.743546499 | 0.035563548 | 0.108703675 |
| rs6679677 | 1 | 114303808 | A | C | 0.224187031 | 0.118821293 | 0.097757518 |
| rs6702421 | 1 | 197559324 | T | C | 0.228973411 | 0.145198468 | 0.120696227 |
| rs6738394 | 2 | 219110625 | A | G | 0.69999953 | 0.054025824 | 0.140209877 |
| rs6738490 | 2 | 234161583 | C | T | 0.727371718 | 0.016801967 | 0.048195115 |
| rs6740462 | 2 | 65667272 | A | C | 0.962318684 | -0.006015822 | 0.127334891 |
| rs6827756 | 4 | 123184411 | C | T | 0.482241043 | 0.098778565 | 0.140569496 |
| rs6908425 | 6 | 20728731 | C | T | 0.76010454 | 0.038465977 | 0.125976074 |
| rs7015630 | 8 | 90875918 | C | T | 0.002312338 | -0.462909111 | 0.151929144 |
| rs7085798 | 10 | 101288347 | A | C | 0.497201807 | -0.042443848 | 0.062518641 |
| rs71624119 | 5 | 55440730 | A | G | 0.401079611 | 0.11914807 | 0.141894519 |
| rs7194886 | 16 | 50725193 | T | C | 0.033397429 | -0.103090058 | 0.048461139 |
| rs7236492 | 18 | 77220616 | T | C | 0.5341907 | 0.092312848 | 0.148503278 |
| rs72727394 | 15 | 38847022 | T | C | 0.169710591 | -0.178237579 | 0.129803454 |
| rs727563 | 22 | 41867377 | T | C | 0.01042097 | 0.361464616 | 0.141112312 |
| rs7438704 | 4 | 48363245 | G | A | 0.80863488 | 0.036947912 | 0.15255912 |
| rs7517847 | 1 | 67681669 | G | T | 0.554581334 | 0.019355953 | 0.032756228 |
| rs7608910 | 2 | 61204856 | G | A | 0.514539419 | 0.060526163 | 0.092862058 |
| rs76906269 | 12 | 40607709 | G | A | 0.418853898 | -0.08774132 | 0.108535506 |
| rs7711427 | 5 | 40414886 | C | A | 0.49954002 | 0.031850986 | 0.047171713 |
| rs7773324 | 6 | 382559 | A | G | 0.575244256 | 0.082545972 | 0.147312811 |
| rs7786444 | 7 | 28154384 | T | C | 0.540457564 | 0.090110184 | 0.147209707 |
| rs77981966 | 2 | 43777964 | T | C | 0.05910824 | 0.229392957 | 0.121539944 |
| rs780094 | 2 | 27741237 | C | T | 0.003624814 | 0.274718199 | 0.094434381 |
| rs7848647 | 9 | 117569046 | C | T | 0.207487533 | -0.106151103 | 0.084213208 |
| rs7969592 | 12 | 68579649 | G | A | 0.956101919 | 0.008197864 | 0.148927856 |
| rs79980175 | 5 | 40521892 | C | A | 0.398020792 | 0.097573329 | 0.115449359 |
| rs8127691 | 21 | 45614860 | C | T | 0.226627921 | -0.107817149 | 0.089172078 |
| rs915286 | 13 | 40695992 | A | G | 0.889537951 | 0.022507315 | 0.162052667 |
| rs9264942 | 6 | 31274380 | C | T | 0.083975644 | 0.130641803 | 0.075599825 |
| rs9457247 | 6 | 167392174 | T | C | 0.748133915 | -0.028302726 | 0.088142775 |
| rs9491892 | 6 | 128280358 | G | T | 0.594106449 | -0.058751423 | 0.110249585 |
| rs9494844 | 6 | 137983469 | A | C | 0.436163218 | 0.106808779 | 0.137164958 |
| rs9554587 | 13 | 100040654 | G | A | 0.958025455 | -0.007356333 | 0.139770335 |
| rs9594766 | 13 | 43040043 | A | G | 0.197645628 | 0.194413137 | 0.1509081 |
| rs9889296 | 17 | 32570547 | A | G | 0.927564827 | 0.007687093 | 0.084558027 |

| **Supplementary Table 5.** Summary of genetic variants (n=86) used to estimate the effect of ulcerative colitis on circulating VEGF in MR analyses. | | | | | | | |
| --- | --- | --- | --- | --- | --- | --- | --- |
| **rsid** | **Chr** | **Pos** | **Effect allele** | **Other allele** | **P-value** | **Beta** | **Se** |
| rs10185424 | 2 | 102662888 | G | T | 0.573775404 | -0.065217729 | 0.115942629 |
| rs10460566 | 2 | 25483121 | A | G | 0.219720103 | -0.198123211 | 0.161433727 |
| rs10748783 | 10 | 101285872 | A | C | 0.467058902 | -0.048556653 | 0.066765399 |
| rs10758669 | 9 | 4981602 | A | C | 0.132472028 | 0.118700163 | 0.078900697 |
| rs10761659 | 10 | 64445564 | G | A | 0.646438413 | 0.042620296 | 0.092912245 |
| rs1077773 | 7 | 17442679 | A | G | 0.382811749 | -0.133096348 | 0.152506232 |
| rs10910092 | 1 | 2501516 | G | A | 0.354484477 | -0.115806954 | 0.125071511 |
| rs11083840 | 19 | 47119910 | G | T | 0.778939163 | 0.046288528 | 0.164902881 |
| rs11150589 | 16 | 30482494 | C | T | 0.645389185 | 0.065120391 | 0.14151162 |
| rs111830527 | 1 | 22687173 | A | G | 0.17658291 | -0.181984568 | 0.13466858 |
| rs11229555 | 11 | 58408687 | T | G | 0.180665205 | 0.201615107 | 0.150604056 |
| rs11230563 | 11 | 60776209 | T | C | 0.632464544 | 0.073268271 | 0.153197294 |
| rs11641184 | 16 | 11704651 | A | C | 0.222395326 | 0.170462119 | 0.139702037 |
| rs11676348 | 2 | 219010146 | T | C | 0.950164662 | -0.009404027 | 0.150464425 |
| rs1182188 | 7 | 2869985 | C | T | 0.590258451 | 0.058542025 | 0.108720903 |
| rs12103 | 1 | 1247494 | C | T | 0.592932574 | 0.085376682 | 0.159704617 |
| rs12132349 | 1 | 200875242 | A | T | 0.033251592 | 0.158138755 | 0.074277294 |
| rs12318183 | 12 | 68503836 | A | C | 0.11117806 | 0.11098916 | 0.069676528 |
| rs12718244 | 7 | 50175654 | A | G | 0.935377471 | 0.012538975 | 0.154647362 |
| rs12720356 | 19 | 10469975 | C | A | 0.110521188 | 0.200940768 | 0.125914183 |
| rs12796489 | 11 | 3059360 | A | C | 0.158849771 | 0.134961345 | 0.095788556 |
| rs1297256 | 21 | 16805676 | T | C | 0.649436284 | -0.049475559 | 0.10884623 |
| rs13136827 | 4 | 123171318 | C | T | 0.40842602 | 0.110898456 | 0.134151359 |
| rs13255292 | 8 | 129076573 | T | C | 0.631944607 | -0.075533705 | 0.157693174 |
| rs13430791 | 2 | 43481013 | A | G | 0.306375395 | 0.169049733 | 0.165272085 |
| rs140143 | 22 | 30173109 | T | G | 0.100821502 | 0.228961732 | 0.139535601 |
| rs1517352 | 2 | 191931464 | C | A | 0.802587349 | -0.035997167 | 0.143988666 |
| rs16841904 | 1 | 197701992 | T | C | 0.253977827 | 0.17902293 | 0.156935685 |
| rs17694108 | 19 | 33731551 | A | G | 0.405408945 | 0.108509209 | 0.130419722 |
| rs17780256 | 17 | 70642923 | C | A | 0.848142214 | -0.023399111 | 0.122195357 |
| rs1801274 | 1 | 161479745 | G | A | 0.33081327 | 0.062026028 | 0.063781481 |
| rs1990760 | 2 | 163124051 | T | C | 0.388475515 | -0.109847476 | 0.127376328 |
| rs2274351 | 10 | 104264107 | T | C | 0.522406906 | -0.099862303 | 0.156122755 |
| rs2395022 | 7 | 98750379 | C | A | 0.419267733 | 0.125614478 | 0.155522687 |
| rs2497318 | 10 | 94432000 | T | C | 0.475818853 | 0.114785652 | 0.160979878 |
| rs2516440 | 6 | 31440497 | A | G | 0.408754766 | 0.095214709 | 0.115259911 |
| rs272882 | 5 | 131669161 | T | G | 0.506612143 | -0.059647872 | 0.089814612 |
| rs2836883 | 21 | 40466744 | A | G | 0.832589113 | 0.011446987 | 0.054153055 |
| rs3024493 | 1 | 206943968 | A | C | 0.248773593 | 0.076454627 | 0.066290139 |
| rs34659678 | 6 | 111888540 | T | C | 0.945947542 | 0.007620971 | 0.112409322 |
| rs35223180 | 1 | 8185902 | T | G | 0.180854227 | -0.131912087 | 0.098579463 |
| rs36070529 | 5 | 158619835 | A | G | 0.194331206 | 0.19955509 | 0.153755561 |
| rs3774937 | 4 | 103434253 | C | T | 0.719614271 | -0.042306298 | 0.117853258 |
| rs3776414 | 5 | 10689562 | G | T | 0.473488774 | 0.114926546 | 0.160329626 |
| rs4366152 | 9 | 117564875 | C | T | 0.178680919 | -0.129599322 | 0.096368727 |
| rs4456788 | 21 | 45616324 | A | G | 0.23013934 | -0.128457152 | 0.107047626 |
| rs4656958 | 1 | 160856964 | G | A | 0.369486365 | 0.127402755 | 0.14196307 |
| rs4676410 | 2 | 241563739 | A | G | 0.865704105 | -0.016194674 | 0.095759812 |
| rs4712520 | 6 | 20640871 | C | T | 0.211299547 | 0.188193898 | 0.150555118 |
| rs4728142 | 7 | 128573967 | A | G | 0.880764615 | 0.018562997 | 0.123753314 |
| rs4743820 | 9 | 93928416 | T | C | 0.154524989 | -0.207605273 | 0.145817989 |
| rs4747886 | 10 | 6176166 | T | C | 0.065664366 | 0.281660007 | 0.153017215 |
| rs4795397 | 17 | 38023745 | G | A | 0.440919039 | 0.060063066 | 0.077938979 |
| rs4812833 | 20 | 43068996 | A | G | 0.66542715 | -0.04644592 | 0.107406189 |
| rs483905 | 11 | 96023427 | A | G | 0.910107279 | 0.016472003 | 0.145894883 |
| rs4947328 | 6 | 31561747 | G | A | 0.839531946 | 0.027144975 | 0.134054415 |
| rs4973341 | 2 | 228660362 | T | C | 0.938684739 | -0.012249398 | 0.159242171 |
| rs4976646 | 5 | 176788570 | C | T | 0.133614403 | 0.224691145 | 0.149794097 |
| rs55808324 | 14 | 88444752 | A | G | 0.922070392 | 0.014149942 | 0.144643854 |
| rs56167332 | 5 | 158827769 | A | C | 0.070242329 | -0.148548469 | 0.082055345 |
| rs59418206 | 10 | 35331624 | A | G | 0.434977929 | 0.12100398 | 0.154993861 |
| rs6062496 | 20 | 62329099 | A | G | 0.892505031 | -0.013169678 | 0.097455618 |
| rs6111031 | 20 | 1682037 | T | C | 0.721969213 | -0.022233893 | 0.062484906 |
| rs61893460 | 11 | 76291154 | A | G | 0.065177792 | -0.165952493 | 0.089994138 |
| rs6426833 | 1 | 20171860 | A | G | 0.948794906 | 0.003012217 | 0.046904517 |
| rs6466198 | 7 | 107480126 | T | A | 0.360825571 | 0.079149959 | 0.086616937 |
| rs661054 | 11 | 114430410 | G | A | 0.036823163 | -0.19061654 | 0.091303721 |
| rs6920220 | 6 | 138006504 | A | G | 0.213997921 | 0.115016061 | 0.092557304 |
| rs7240004 | 18 | 46395022 | G | A | 0.978819712 | 0.003642302 | 0.137193364 |
| rs7404095 | 16 | 23864590 | C | T | 0.949716287 | 0.009753569 | 0.154663739 |
| rs7547569 | 1 | 67731368 | C | T | 0.072397529 | -0.085535434 | 0.047609345 |
| rs7608910 | 2 | 61204856 | G | A | 0.514539419 | 0.057435542 | 0.088120284 |
| rs76546301 | 7 | 50498389 | A | G | 0.686392252 | -0.064915459 | 0.160778986 |
| rs76904798 | 12 | 40614434 | T | C | 0.697358342 | 0.060215629 | 0.15484019 |
| rs7711427 | 5 | 40414886 | C | A | 0.49954002 | 0.088825924 | 0.131552317 |
| rs7738430 | 6 | 31508836 | C | T | 0.954575445 | 0.004891783 | 0.085877966 |
| rs79045992 | 16 | 68518992 | A | G | 0.342898579 | -0.155809404 | 0.164277307 |
| rs8096327 | 18 | 12887750 | G | A | 0.248019829 | 0.142848005 | 0.123659467 |
| rs913678 | 20 | 48955424 | C | T | 0.519531472 | 0.100319571 | 0.155759333 |
| rs9271255 | 6 | 32580357 | T | C | 0.297883157 | 0.044567815 | 0.042813177 |
| rs9271858 | 6 | 32595223 | G | A | 0.730223993 | 0.032597446 | 0.094532593 |
| rs941823 | 13 | 41013977 | C | T | 0.683273675 | 0.046931508 | 0.115028205 |
| rs9611131 | 22 | 39662480 | C | T | 0.108763969 | 0.178701575 | 0.111425688 |
| rs9836291 | 3 | 49697459 | A | G | 0.694199544 | -0.027017979 | 0.068719641 |
| rs9891119 | 17 | 40507980 | C | A | 0.379233028 | -0.113907045 | 0.129541345 |
| rs9941524 | 2 | 199499443 | G | A | 0.806104499 | 0.027631207 | 0.112571585 |
